# Risk Factors for Recent Suicide Attempts in Major Depressive Disorder Patients in China: Results From a National Study

**DOI:** 10.3389/fpsyt.2018.00300

**Published:** 2018-07-03

**Authors:** Li-Min Xin, Lin Chen, Yun-Ai Su, Fu-De Yang, Gang Wang, Yi-Ru Fang, Zheng Lu, Hai-Chen Yang, Jian Hu, Zhi-Yu Chen, Yi Huang, Jing Sun, Xiao-Ping Wang, Hui-Chun Li, Jin-Bei Zhang, Tian-Mei Si

**Affiliations:** ^1^Key Laboratory of Mental Health, Ministry of Health (Peking University), National Clinical Research Center for Mental Disorders (Peking University Sixth Hospital), Peking University Sixth Hospital and Peking University Institute of Mental Health, Beijing, China; ^2^Beijing Hui-Long-Guan Hospital, Peking University, Beijing, China; ^3^Mood Disorders Center, Beijing Anding Hospital, Capital Medical University, Beijing, China; ^4^Division of Mood Disorders, Shanghai Mental Health Center, Shanghai Jiao Tong University School of Medicine, Shanghai, China; ^5^Shanghai Tongji Hospital, Tongji University Medical School, Shanghai, China; ^6^Division of Mood Disorders, Shenzhen Mental Health Centre, Shenzhen, China; ^7^The First Hospital of Harbin Medical University, Harbin, China; ^8^Hangzhou Seventh People's Hospital, Hangzhou, China; ^9^West China Hospital, Sichuan University, Chengdu, China; ^10^The Affiliated Brain Hospital, Nanjing Medical University, Nanjing, China; ^11^Mental Health Institute, The Second Xiangya Hospital, Central South University, Changsha, China; ^12^The Second Affiliated Hospital, College of Medicine, Zhejiang University, Hangzhou, China; ^13^The Third Affiliated Hospital of Sun Yat-Sen University, Guangzhou, China

**Keywords:** suicide attempt, major depressive disorder, risk factor, melancholic features, China

## Abstract

**Objective:** To analyze the factors associated with recent suicide attempts including socio-demographic and clinical characteristics in major depressive disorder (MDD) patients in China.

**Methods:** The data were from a nationwide sample from 13 major psychiatric hospitals or the psychiatric units of general hospitals in China, from September 1, 2010 to February 28, 2011. Melancholic features and suicide attempts in the past month were defined according to the melancholic feature module and the suicide module of the Mini International Neuropsychiatric Interview (MINI). Socio-demographic and clinical characteristics were compared between MDD patients with and without recent suicide attempts. Further analyses regarding the factors associated with recent suicide attempts in MDD patients were performed via multivariate logistic regression analysis.

**Results:** Among 1,172 MDD patients, 57 (4.9%) were reported to have made a suicide attempt in the past month. Compared to the MDD patients without recent suicide attempt, significantly higher percentage of patients in the recent suicide attempters group had previous suicide attempts (χ^2^ = 171.861, *p* < 0.001) and depressive episodes with melancholic features (χ^2^ = 22.837, *p* < 0.001). Logistic regression analysis indicated that previous suicide attempts (*OR* = 20.81, 95% CI: 11.12–38.94, *p* < 0.001) and depressive episodes with melancholic features (*OR* = 4.43, 95% CI: 2.09–9.43, *p* < 0.001) were independently associated with recent suicide attempts in MDD patients.

**Limitations:** Cross-sectional design, retrospective recall of suicide attempt data.

**Conclusion:** Recent suicide attempts are associated with melancholic features and previous suicide attempts in MDD patients in China. These data may help clinicians to identify MDD patients at high risk of suicide attempt behavior.

## Introduction

Suicide is a serious public health problem that has received increasing attention worldwide (1, 2). According to a WHO report, an estimated 7,88,000 people died from suicide in the year 2015 globally, and the age-standardized suicide rate was 10.7 per 1,00,000 persons (3). In China, the suicide rate was 8.5 per 1,00,000 persons (3).

A national case-control psychological autopsy study conducted in China showed that mental disorders are present in up to 63% of people who died by suicide (4). The most common mental illness among the individuals who died by suicide was major depressive disorder (MDD) (4). A Swedish epidemiological study showed that depression is associated with a 20-fold increased risk of suicide (5). There is compelling evidence, however, that adequate prevention and management of suicidal behavior among MDD patients is beneficial to reduce suicide rates (6). A previous suicide attempt is the strongest risk factor for subsequent suicide attempt and death (7–9). Identifying the factors associated with suicide attempts is important for early identification efforts and can lead to reduction in the incidence of suicide.

Previous studies in Western countries reported risk factors for suicide attempts in MDD patients. They include family history of psychiatric disorders (10), adverse life events such as unemployment and divorce (11), comorbidity with anxiety disorders (12), early age at onset (13, 14), more frequent depressive episodes (14) and higher number of admissions (14). Because of different socio-cultural and economic contexts between the Western countries and China, these findings may not necessarily apply to the Chinese population (15).

There has been limited study of the risk factors for attempted suicide in MDD patients in China. A case-control study of 215 suicide attempters with MDD showed that hopelessness, negative life-events and family history of suicide were risk factors for attempted suicide (16). The small sample size and single study site limit the generalizability of the findings. Another study of 6,008 Han Chinese women diagnosed with recurrent MDD showed that more depressive symptoms, stressful life events, positive family history of MDD, a greater number of episodes, significant melancholic symptoms, and earlier age of onset were risk factors for suicide (17). However, this study was limited to female MDD patients, so it does not necessarily pertain to both genders.

The Diagnostic Assessment Service for People with Bipolar Disorder in China (DASP) is the first nationwide, multicenter and large sample study conducted in patients with major affective disorders in China. A previous study using this data analyzed factors associated with suicide risk in MDD patients including social-demographic and clinical features (18). The following risk factors were identified: male gender, unemployment, more frequent depressive episodes, depressive episodes with suicidal ideation, and attempts, depressive episodes with psychotic symptoms, and no current antidepressant use. Previous suicide attempts has been identified as one of the best predictor of eventual suicide death (19). A 5-year follow-up study showed that 16–34% of subjects attempted suicide again within 1 or 2 years after their first attempt (20). In order to find risk factors associated with recent a suicide attempt, therefore, we re-analyzed the data of the DASP project. The goal was to see if we could identify a group of patients with high risk of suicide attempt behaviors for whom it would be appropriate to provide more aggressive clinical management and effective suicide prevention measures.

## Methods

### Study participants and settings

The data were obtained from DASP (21–23), which was initiated by the Chinese Society of Psychiatry and conducted in 13 major psychiatric hospitals or the psychiatric units of general hospitals from September 1, 2010 to February 28, 2011. In- and outpatients who received treatment and met the following criteria were enrolled: (i) aged 16–65 years old; (ii) DSM-IV or ICD-10 diagnosis of MDD based on a review of medical records; (iii) understood the purpose of the study and provided written informed consent. Exclusion criteria were as follows: (i) a history of or ongoing severe somatic diseases, such as severe cardio-cerebral vascular diseases, respiratory diseases, liver diseases, kidney diseases, or malignant tumors; (ii) depressive disorders secondary to a general medical or neurological condition; (iii) a previous diagnosis of bipolar disorder; (iv) treatment with electroconvulsive therapy in the past month. The study protocol was approved by the Ethics Committees of all participating centers.

### Instrument and assessment procedures

Patients with MDD who were receiving treatment in the participating hospitals/units were consecutively referred by their treating psychiatrists to the research team to be screened for eligibility. Patients fulfilling the study entry criteria were invited to participate in the study (22). The patients' basic socio-demographic and clinical characteristics were collected with a questionnaire designed for the study. The items of questionnaire included gender, marital status, employment status, education level, frequent depressive episodes, symptom characteristics of depressive episodes, major depressive episodes following obvious causes (such as postpartum); seasonal depressive episodes; family history of psychiatric disorders; age, age at onset, number of lifetime depressive episodes, number of admissions, receiving antidepressants treatment and other medicines. Frequent depressive episodes were defined as more than 4 depressive episodes in the previous year (24). Assessment of symptom characteristics of depressive episodes including atypical depressive features (increased appetite, increased sleep, and weight gain), psychotic symptoms, anxiety symptoms, or disorder and seasonal depressive episodes was completed by a clinical interview and was supplemented by a review of patients' medical records.

Prior to the study, all 13 raters (qualified psychiatrists) of research team were trained in the use of the Chinese version of the Mini International Neuropsychiatric Interview (MINI), Version 5.0 (25, 26) in 20 patients treated for MDD. The kappa values were greater than 0.85. After providing written consent following a full explanation about the study, patients met a rater for a confirmatory diagnostic interview of MDD based on DSM-IV criteria using the MINI supplemented by a review of medical records and, whenever possible, an interview of relatives.

#### Recent suicide attempts and previous suicide attempts

Recent suicide attempts were assessed by one of the questions in the suicidal module of MINI (21). “Did you attempt suicide within the past month?” If the question was answered “yes,” the patient was classified as a recent suicide attempter. Previous suicide attempts were also assessed by one of the questions in MINI (21). “In your lifetime (the past month was not included), did you ever make a suicide attempt? The responses of patients were recorded.

#### Melancholic features

Melancholic features were defined using the melancholic features module of MINI, which is in accordance with the DSM-IV criteria (27). The criteria include loss of pleasure in all, or almost all activities and/or lack of reactivity to usually pleasurable stimuli, plus three (or more) features of the following: distinct quality of depressed mood, depression regularly worse in the morning, early morning awakening (at least 2 h before usual awakening), marked psychomotor retardation or agitation, significant anorexia or weight loss, and excessive or inappropriate guilt.

### Statistical analysis

The data were analyzed using SPSS, version 20.0 (SPSS Inc., Chicago, IL, USA). Group differences between the recent suicide attempters and a non-suicide attempters control group were measured using independent-sample *t*-tests or Mann-Whitney *U*-tests for continuous data and chi-square tests for categorical data. Multiple logistic regression analysis was performed to identify the factors associated with recent suicide attempt in MDD patients. The variables with a univariate *p*-value < 0.05 and possible clinical variables related to suicide such as age, gender, depressive episodes with psychotic symptoms, and depressive episodes with anxiety symptoms or disorder were included in the multiple logistic regression analysis. The level of significance was set at 0.05 (two-tailed).

## Results

### Patients

The total number of screened patients was 1,757. Of these patients, 270 patients refused or failed to complete the interview, 306 patients were excluded because of meeting DSM-IV criteria for bipolar disorder based on the MINI, and 9 patients were lacking information on the suicidal module of the MINI. Ultimately, 1,172 patients were included in the present study, and 57 (4.9%) of the subjects had a suicide attempt during the past month.

### Basic socio-demographic and clinical features

Table 1 shows the comparison of the socio-demographic and clinical characteristics between recent suicide attempters and the non-suicide attempters group. Compared to the MDD patients without recent suicide attempt, significantly higher percentage of patients in the recent suicide attempters group had previous suicide attempts (χ^2^ = 171.861, *p* < 0.001) and depressive episodes with melancholic features (χ^2^ = 22.837, *p* < 0.001).

**Table 1:** Comparison of the basic demographic and clinical variables between recent suicide attempters and non-recent suicide attempters diagnosed with MDD.

**Item**	**Total**		**Non-suicide attempters**		**Suicide attempters**				
	**(*****n*** = **1,172)**		**(*****n*** = **1,115)**		**(*****n*** = **57)**		**Statistics**		
	***N***	**%**	***N***	**%**	***N***	**%**	**χ^2^**	***df***	***p***
Female	789	67.3	748	67.1	41	71.9	0.579	1	0.447
Married	821	70.1	780	70	41	71.9	0.101	1	0.751
Unemployed	557	47.5	531	47.6	26	45.6	0.088	1	0.767
Education (senior secondary school and below)	664	56.7	631	56.6	33	57.9	0.037	1	0.846
Frequent depressive episodes (> 4 in the previous year)	89	7.6	82	7.4	7	12.3	1.876	1	0.171
Depressive episodes with									
Increased appetite and sleep and weight gain	179	15.3	169	15.2	10	17.5	0.239	1	0.625
Psychotic symptoms	156	13.3	146	13.1	10	17.5	0.931	1	0.335
Anxiety symptoms or disorder	910	77.6	869	77.9	41	71.9	1.127	1	0.288
Melancholic features	626	53.4	578	51.8	48	84.2	22.837	1	<0.001
Previous suicide attempts	168	14.3	126	11.3	42	73.7	171.861	1	<0.001
Seasonal depressive episodes	133	11.3	127	11.4	6	10.5	0.04	1	0.841
Family history of psychiatric disorders	203	17.3	197	17.7	6	10.5	1.931	1	0.165
Any use of antidepressants	816	69.6	776	69.6	40	70.2	0.009	1	0.926
Any use of antipsychotics	225	19.2	211	18.9	14	24.6	1.111	1	0.292
Any use of mood stabilizers	44	3.8	40	3.6	4	7.0	1.766	1	0.184
Any use of benzodiazepines	191	16.3	179	16.1	12	21.1	0.993	1	0.319
	**Mean**	**SD**	**Mean**	**SD**	**Mean**	**SD**	***T*****/*****Z***		***P***
Age (years)	40.5	12.8	40.7	12.9	39	13	0.899	1,170	0.369^⋆^
Age at onset (years)	34.7	12.5	35	12.5	34.6	12.3	0.028	1,170	0.978^⋆^
Lifetime depressive episodes	1.9	2.6	1.9	2.6	1.4	1.6	−1.553	–	0.121^Δ^
Number of admissions	0.4	1	0.4	1	0.6	1.1	−1.93	–	0.054^Δ^

*n, Number; SD, standard deviation*.

⋆ Independent-sample t- tests.

Δ *Mann-Whitney U-test*.

### Correlates of recent suicide attempts

Candidate variables including previous suicide attempts and depressive episodes with melancholic features with a *p*-value < 0.05 on univariate analysis and clinical relevant variables including age, gender, depressive episodes with psychotic symptoms, depressive episodes with anxiety symptoms, or disorder were entered into multivariable model. Multiple logistic regression analysis with the forward stepwise method also showed that previous suicide attempts (*OR* = 20.81, 95% CI: 11.12–38.94, *p* < 0.001) and depressive episodes with melancholic features (*OR* = 4.43, 95% CI: 2.09–9.43, *p* < 0.001) were the risk factors for recent suicide attempts in MDD patients (Table 2).

**Table 2:** Multiple logistic regression analysis of the risk factors for recent suicide attempts in MDD patients.

**Risk factor (yes = 1, no = 0)**	**β**	***S*.*E***.	***Waldχ*^2^**	**OR**	**95% CI**	***p*-value**
Previous suicide attempts	3.04	0.32	90.20	20.81	11.12–38.94	<0.001
Depressive episodes with melancholic features	1.49	0.39	14.98	4.43	2.09–9.43	<0.001

*OR, odds ratio; CI, confidence interval*.

## Discussion

Among 1,172 treatment-seeking outpatients and inpatients with MDD, 4.9% of the subjects had suicide attempt behavior in the past month. The patients with recent suicide attempt behaviors were more likely to have melancholic features and previous suicide attempts.

In the present study, 14.3% of the patients with MDD had previous suicide attempts. Previous Western studies had suggested the important effect of previous suicide attempts for future suicides (8). Among the previous suicide attempters in this study, 25% of them made a suicide attempt during the investigated time. Therefore, patients with previous suicide attempts should be carefully monitored. It is necessary to provide them with additional support and services by clinicians and other care providers.

Previous studies also have proposed that melancholic features may be associated with past suicide attempts and the probability of future attempts (28, 29). A more recent study in six Asian countries showed that the suicidal risk in MDD patients with melancholic features is 1.79 times higher than in subjects without melancholic features (30). Another study that investigated Chinese women with MDD also showed that melancholia is associated with suicide attempts (17). Consistent with these studies, we also found that melancholic features is a risk factor for suicide attempt in the investigated patients. Numerous studies have documented that melancholic depression is associated with poorer treatment response, more severe cognitive impairment, greater severity of illness, and higher suicide risk than MDD patients without melancholic features (30–34). These help explain the high suicide attempt rate in MDD patients with melancholic features.

Several studies showed that psychotic symptoms increased the risk of suicide in MDD patients (35, 36). One 4-year follow up study conducted in Finland found that psychotic symptoms increased the risk of completed suicide after a serious suicide attempt by 3.32 fold compared with patients without psychotic features (37). However, others have not confirmed this finding (38). A systematic review showed that psychotic symptoms increase the risk of suicide attempts during an acute episode of MDD, but they have little effect on attempted suicide after the acute episode is remitted (39). Psychosis was not identified as an independent risk factor for recent suicide attempts in the present study. This may be because many subjects were not in an acute episode at the time of evaluation.

Most previous studies conducted in Western countries reported that women make more suicide attempts but more males die due to their suicide attempts (13, 40). In China the pattern was different: more women than men make suicide attempts and complete suicides (41). This was the case according to data before the year 2000. However, no gender difference was found after the year 2000 in China (42). In the investigated group, women constituted about 75 percent of recent suicide attempters. However, no association was found between suicide attempts and gender.

There are several limitations in the present study. First, the DASP was a cross-sectional survey in which most of the data were retrospectively collected. Therefore, recall bias might have affected the accuracy of the data. Second, the cross-sectional nature of the study could not establish a causal relationship between the demographic and clinical features and the suicide attempts. A prospective study should be carried to evaluate risk factors of suicide in patients diagnosed with MDD. Third, the severity of depression in patients with MDD was not evaluated. Perroud et al. found that MDD patients with suicide attempts were more severely ill than subjects who had not attempted suicide (43). The Sequenced Treatment Alternatives to Relieve Depression (STAR^*^D) study also found that subjects with prior suicide attempts had greater current severity of medical illness than non-attempters in MDD patients (13). However, a 10-year longitudinal study did not find an association between severity of depression symptoms and future suicide attempt (12). Fourth, as the investigation of suicide variables was not a primary aim of the DASP project, other potential risk factors for suicide attempts including misuse of alcohol and drugs (10) and co-morbid personality disorders (12, 44, 45) were not recorded. The present study, however, has a multi-center design and a large sample size and therefore may reflect the real world of clinical practice with MDD patients in China.

In conclusion, two clinical features were found to be related to recent suicide attempt in MDD patients in our study. Focusing on these clinical characteristics may help clinicians to identify the patients with high-risk of suicidal behavior and implement effective preventive interventions to reduce suicide.

## Author contributions

Y-AS and T-MS designed research. F-DY, GW, Y-RF, ZL, H-CY, JH, Z-YC, YH, JS, X-PW, H-CL, and J-BZ performed research and collected data. L-MX and LC analyzed data. L-MX, Y-AS, and T-MS wrote the manuscript.

### Conflict of interest statement

The authors declare that the research was conducted in the absence of any commercial or financial relationships that could be construed as a potential conflict of interest.
